# Intracranial complications in adult patients with severe pneumococcal meningitis: a retrospective multicenter cohort study

**DOI:** 10.1186/s13613-024-01405-z

**Published:** 2024-12-19

**Authors:** Camille Legouy, Renaud Cornic, Keyvan Razazi, Damien Contou, Stéphane Legriel, Eve Garrigues, Pauline Buiche, Maxens Decavèle, Sarah Benghanem, Thomas Rambaud, Jérôme Aboab, Marina Esposito-Farèse, Jean-François Timsit, Camille Couffignal, Romain Sonneville

**Affiliations:** 1 Service d’anesthésie-réanimation, GHU Paris Psychiatrie & Neurosciences, Paris, France; 2 Centre d’Investigation Clinique, Hôpital Bichat Claude Bernard, AP-HP, Hôpital Bichat, Inserm CIC 1425, 46, rue Henri Huchard, Paris, 75018 France; 3 Département d’Epidémiologie, Biostatistique et Recherche, AP-HP, Hôpital Bichat, Paris, 75018 France; 4 Médecine intensive réanimation, Hôpital Henri Mondor, Créteil, France; 5 Médecine intensive réanimation, Centre hospitalier d’Argenteuil, Argenteuil, France; 6 Médecine intensive réanimation, Centre hospitalier de Versailles, Le Chesnay, France; 7 Médecine intensive réanimation, Hôpital Ambroise Paré, Boulogne-Billancourt, France; 8 Médecine intensive réanimation, Hôpital de Saint Antoine, Paris, France; 9 Groupe Hospitalier Universitaire APHP-Sorbonne Université, site Pitié-Salpêtrière, Service de Médecine Intensive et Réanimation (Département R3S), Paris, F-75013 France; 10 Sorbonne Université, INSERM, UMRS1158 Neurophysiologie Respiratoire Expérimentale et Clinique, Paris, F-75005 France; 11 Médecine intensive réanimation, Hôpital Cochin, Paris, France; 12 Médecine intensive réanimation, Hôpital Avicenne, Bobigny, France; 13 Médecine intensive réanimation, Hôpital Delafontaine, Saint-Denis, France; 14 Université Paris Cité, INSERM U1137, Paris, F-75018 France; 15 Médecine intensive réanimation, Hôpital Bichat - Claude Bernard, 6 Rue Henri Huchard, Paris, 75018 France; 16 Université Paris Cité, IAME, INSERM UMR 1137, Paris, 75018 France

**Keywords:** Pneumococcal meningitis, Intracranial complications, Neurology, Intensive care unit, Neuropronostication

## Abstract

**Background:**

We aimed to investigate the association of intracranial complications diagnosed on neuroimaging with neurological outcomes of adults with severe pneumococcal meningitis.

**Methods:**

We performed a retrospective multicenter study on consecutive adults diagnosed with pneumococcal meningitis requiring at least 48 h of stay in the intensive care unit (ICU) and undergoing neuroimaging, between 2005 and 2021. All neuroimaging were reanalyzed to look for intracranial complications which were categorized as (1) ischemic lesion, (2) intracranial hemorrhage (3) abscess/empyema, (4) ventriculitis, (5) cerebral venous thrombosis, (6) hydrocephalus, (7) diffuse cerebral oedema. The primary outcome was unfavorable outcome at 90 days after ICU admission, defined by a modified Rankin Scale (mRS) score > 2.

**Results:**

Among the 237 patients included, intracranial complications were diagnosed in 68/220 patients (31%, 95%CI 0.25–0.37) who underwent neuroimaging at ICU admission and in 75/110 patients (68%, 95%CI 0.59–0.77) who underwent neuroimaging during ICU stay. At 90 days, 103 patients (44%, 95%CI 37–50) had unfavorable outcome, including 71 (30%) deaths. The most frequent intracranial complications were ischemic lesion (69/237 patients, 29%), diffuse cerebral oedema (43/237, 18%) and ventriculitis (36/237, 15%). Through multivariable analysis, we found that intracranial complications (adjusted odds ratio (aOR) 2.88, 95%CI 1.37–6.21) were associated with unfavorable outcome, along with chronic alcohol consumption (aOR 3.10, 95%CI 1.27–7.90), chronic vascular disease (aOR 4.41, 95%CI 1.58–13.63), focal neurological sign(s) (aOR 2.38, 95%CI 1.11–5.23), and cerebrospinal fluid leukocyte count < 1000 cell/microL (aOR 4.24, 95%CI 2.11–8.83). Competing risk analysis, with persistent disability (mRS score 3–5) as the primary risk and ICU-death as the competing risk, revealed that chronic alcohol consumption was the sole significant variable associated with persistent disability at 90 days (cause-specific hazard ratio 4.26, 95%CI 1.83–9.91), whereas the remaining variables were associated with mortality.

**Conclusions:**

In adults with severe pneumococcal meninigitis, intracranial complications were independently associated with a higher risk of poor functional outcome, in the form of persistent disability or death. This study highlights the value of neuroimaging studies in this population, and provides relevant information for prognostication.

**Supplementary Information:**

The online version contains supplementary material available at 10.1186/s13613-024-01405-z.

## Introduction

Pneumococcal meningitis is the most common cause of community-acquired bacterial meningitis in adult patients, accounting for 54 to 70% of cases [1–3], and is associated with the highest morbidity and mortality rates [2, 4–7]. It is associated with acute intracranial complications at disease onset or later during the course of the disease, in the form of ischemic stroke (14–36% of cases) [5, 8], cerebral oedema (11–29%) [10, 11], cerebral hemorrhage (1–3%) [10, 12] and cerebral venous thrombosis (1–9%) [10, 11, 13, 14]. Other less frequent complications include infectious complications (such as abscess, empyema, ventriculitis) in less than 5% of cases [9, 14–16] and hydrocephalus (3–6% of cases) [9, 17, 18].

Intracranial complications likely contribute to a more severe presentation and poorer outcomes, including increased short-term mortality, and persistent disability in survivors. However, few studies evaluated the prevalence and prognostic significance of these intracranial complications, based on routine neuroimaging diagnostic studies [14, 19, 20]. As a result, recommendations for the diagnosis and management of those complications are limited [17, 21, 22].

In the present study, we aimed to evaluate the prevalence of intracranial complications diagnosed on neuroimaging and their association with neurological outcomes at 90 days of adult patients with severe pneumococcal meningitis, requiring care in the intensive care unit (ICU).

## Materials and methods

### Study Design

We performed a retrospective multicenter study in 11 centers in the greater Paris area, France, between February 2005 and September 2021. We included consecutive adult patients with pneumococcal meningitis who required at least 48 h of ICU stay and who underwent neuroimaging studies at admission and/or during ICU stay. The study protocol PNEUMATICS was approved for all participating sites by the French regulatory authorities CNIL (Commission Nationale de l’Informatique et des Libertés, n°922134) on June 30, 2022 and the local ethic committee (CLEA-2021-209). Procedures were followed in accordance with the ethical standards of the responsible committee on human experimentation (institutional or regional) and with the Helsinki Declaration of 1975.

### Patients

Patients were identified by the Medical Information Department of each participating center, based on the following International Classification of Diseases 10th Revision (ICD-10) codes: G00.9, G00.1, G00.2, and G00.8. To be included, patients had to meet all the following inclusion criteria: (1) age ≥ 18 years; (2) an admission to the ICU; (3) a diagnosis of pneumococcal meningitis at ICU admission. Pneumococcal meningitis was defined by at least one of the following: (a) a cerebrospinal fluid (CSF) culture positive for *S. pneumoniae*; (b) the combination of CSF pleocytosis with a positive blood culture for *S.pneumoniae* [2, 9]; 4) Brain imaging performed within 30 days following ICU admission. Exclusion criteria were (1) a duration of ICU stay < 48 h; (2) no brain imaging performed.

### Clinical and biological data

Patients’ medical history, clinical and biological data, and treatments were retrieved from electronic medical charts and collected on a secured electronic case record form (REDCap software). Chronic alcohol consumption was defined as consumption of more than 10 glasses of alcohol a week. If the patient was nonconscious enough to respond to the intensivist, data of complications of chronic alcohol use were search in the database. The Simplified Acute Physiology Score (SAPS) 2 and the Glasgow Coma Scale (GCS) were collected at admission [23, 24]. The score on the Glasgow Coma Scale was classified into three categories: < 8 (comatose), ≥ 8 and < 14 (altered mental status) or ≥ 14 (normal) [5]. Signs of shock were defined as skin mottling with cold extremities and septic shock was defined as persistent hypotension requiring norepinephrine infusion and elevated lactate levels ≥ 2 mmol/l [25]. Immunosuppression was defined by history of cancer <5 years, HIV infection, chronic use (> 3 months) of immunosuppressive drugs or splenectomy. Body temperature was classified into three categories: (1) ≤36 °C (hypothermia), (2) >36 °C and ≤38 °C (normothermia), (3) >38 °C (fever). CSF leukocytes count was dichotomized as ≥ 1000 or <1000 cells/microL and CSF glucose as ≥2.5 or <2.5 mmol/l.

### Neuroimaging

All available neuroimaging studies were reinterpreted by an experienced neurologist (CL) blinded to patients’ outcomes and to radiologist’s interpretation. All CT scan and MR studies were retrieved from the electronic radiological database of the participating centers. The following intracranial complications were evaluated: (1) ischemic lesion, (2) intracranial hemorrhage (i.e., intracerebral hematoma, subdural hematoma, or subarachnoid hemorrhage), (3) abscess/empyema, (4) ventriculitis, (5) cerebral venous thrombosis, (6) hydrocephalus, and (7) diffuse cerebral oedema (Additional eTable 1). Three time periods were considered to describe the timeline of intracranial complications namely, at ICU admission (i.e., the first available neuroimaging at hospital including emergency department or on the day after ICU admission), during ICU stay, and regardless of the time of hospitalization (including neuroimaging at ICU discharge). A new intracranial complication was noted by binary score (1/0) for each new complication diagnosed between 2 neuroimaging studies.

### Outcomes

Outcome at 90 days was scored on the modified Rankin Scale (mRS) based on information retrieved from follow-up consultations. We defined unfavorable outcome as mRS score > 2, indicating moderate to severe disability or death. Patients discharged within 90 days with a disability had their charts reviewed and were classified according to the latest available date. Patients discharged from hospital within 90 days following ICU admission without any disability were considered to have a favorable outcome. Causes of death were categorized as neurological (including brain herniation, neurological complication, brain death, withdrawal of care due to an expected poor neurological prognosis) or systemic (including septic shock, cardiorespiratory failure, multi-organ dysfunction syndrome or other systemic complication) causes by investigators.

### Statistical methods

Data are described as median (interquartile range) or numbers (frequency) for continuous and categorical variables, respectively. All intracranial complications were grouped under one unique variable for prognostic analyses. Variables with more than 10% of missing data were excluded from models.

A model was fitted on the predictive factors at admission in ICU to assess the unfavorable neurological outcome at 90 days (mRS > 2) by means of a multivariate logistic regression. To construct the model, variables clinically identified by previously published data were selected in univariate analyses with the threshold of p-value ≤ 0.15 on Wald test. All selected variables were entered into a multivariable logistic model, with the dichotomized mRS as the dependent variable. The final model was selected by backward stepwise methods using the AIC criteria. Discrimination of the final model was evaluated using C-statistic and the calibration (goodness of fit) by Hosmer-Lemeshow and cross-validation by bootstrap (500 samples).

Due to the incidence of death in this ICU population, we performed a competing risk analysis, with the ICU-death as the competing risk and the unfavorable functional outcome (mRS 3–5) as the risk of interest. Cause-specific hazard ratios (CSHRs) for unfavorable functional outcome were modeled in multivariate analysis by means of Cox regressions to estimate the effects of the risk factors on the hazard ratios of unfavorable functional outcome in the presence of the ICU-death as a competing risk. Sub distribution hazard (SHRs) were modeled using Fine and Gray methods [26] to assess the effect of risk factors on the cumulative incidence function (CIF) on the unfavorable neurological outcome in the presence of the risk of death on unfavorable functional outcome [27].

Analyses were performed using R version 4.1.2 (The R Foundation for Statistical Computing; www.r-project.org/) [28]. Our methodology and results were presented according to STROBE recommendations [29].

## Results

### Patients

Among 413 patients identified, 237 fulfilled the eligibility criteria and were included in the study. A flowchart is presented in Additional eFigure 1. A total of 173 patients were excluded, including 79 patients who stayed in ICU less than 48 h, and 94 without neuroimaging during ICU stay.

Baseline characteristics of the 237 patients included in the study are described in Table 1. Half the patients were male (54%) with a median age of 60 years [49–69]. A history of diabetes was present in 48 patients (20%), 57 patients (24%) were immunocompromised, chronic alcohol consumption was present in 48 patients (20%) whereas a history of head trauma were noted in 18 patients (8%). Chronic vascular disease was recorded in 35 patients (15%). At ICU admission, patients were severely ill, as reflected by a SAPSII of 42 [31–58]. Half of them presented with altered mental status (score on the GCS between 8 and 13) and 30% were comatose (score on the GCS<8). Focal neurological signs were noted in 63 patients (27%), and seizures were present in 39 patients (16%). Nearly half of the patients had CSF count < 1000 cell/microL, a median CSF protein of 4.9 [3.0–7.2] g/l and three quarters of patients had CSF glucose < 2.5 mmol/l.

**Table 1:** Baseline characteristics

	All patients *N* = 237	mRS 0–2 *N* = 133	mRS 3–6 *N* = 103	*P* Value
**Demographics**				
Age (years)	60 [49–69]	60 [49–69]	61 [50–70]	0.37
Male sex	129 (54)	69 (52)	60 (58)	0.33
**Medical history**				
Diabetes	48 (20)	26 (20)	22 (21)	0.86
Alcoholism	48 (20)	21 (16)	28 (27)	0.07
Immunosuppression	57 (24)	29 (22)	28 (27)	0.42
Head trauma	18 (8)	13 (10)	5 (5)	0.24
Recent NSAID use	36 (15)	19 (14)	17 (17)	0.77
Chronic vascular disease	35 (15)	13 (10)	22 (21)	0.02
Long term use of anticoagulant	11 (5)	4 (3)	7 (7)	0.18
**Clinical presentation at admission**				
SAPS 2	42 [31–58]	37 [27–48]	53 [39–66]	< 0.01
Systolic blood pressure (mmHg)^a^	130 [110–155]	137 [118–157]	128 [107–149]	0.06
Diastolic blood pressure (mmHg)^b^	75 [60–87]	76 [64–90]	72 [59–83]	0.01
Signs of shock	68/237 (29)	21/133 (16)	46/103 (45)	< 0.01
Temperature (°C)^c^				0.01
≤ 36 °C	18/220 (8)	5/124 (4)	13/95 (14)	
> 36 °C and ≤ 38 °C	101/220 (46)	58/124 (47)	42/95 (44)	
> 38 °C	101/220 (46)	61/124 (49)	40/95 (42)	
Glasgow coma score^d^				< 0.01
≥ 14	45/228 (20)	31/127 (24)	14/100 (14)	
≥ 8 and < 14	115/228 (50)	73/127 (57)	41/100 (41)	
< 8	68/228 (30)	23/127 (18)	45/100 (45)	
Focal neurological signs	63 (27)	26 (20)	36 (35)	0.01
Seizures	39 (16)	16 (12)	23 (22)	0.05
**Blood and CSF findings**				
CSF leukocyte count^e^				
cell / microL	970 [101–3500]	1500 [350–6339]	430 [42–1555]	0.19
< 1000 cell/microL	108/214 (46)	45/122 (37)	63/91 (69)	< 0.01
% of neutrophils	92 [85–96]	94 [89–97]	90 [80–96]	0.02
Positive CSF Culture^f^	202/235 (86)	111/132 (84)	90/102 (88)	0.48
CSF protein (g/l)^g^	4.9 [3.0–7.2]	4.8 [3.0–6.6]	5.1 [3.1–8.0]	0.20
CSF glucose < 2.5 mmol/l^h^	156/202 (77)	83/114 (73)	72/88 (82)	0.14
Positive blood culture^i^	126/209 (60)	62/117 (53)	64/92 (70)	0.022
**Causes of meningitis**				< 0.01
Pneumonia	41 (17)	18 (14)	23 (22)	
Otitis or sinusitis	98 (41)	69 (52)	29 (28)	
Cerebrospinal fluid leak	12 (5)	11 (8)	1 (1)	
Endocarditis	7 (3)	0 (0)	7 (7)	
Other^j^	6 (3)	4 (3)	2 (2)	
Unknown	73 (31)	31 (23)	42 (41)	

Data are median [Interquartile Range] or number/N (%)

*mRS* modified Rankin Scale; *SAPS II* Simplified Acute Physiology Score; *CSF* cerebrospinal fluid; *NSAID* nonsteroidal anti-inflammatory drugs

^a^Systolic blood pressure was determined in 214 patients

^b^Diastolic blood pressure was determined in 213 patients

^c^Temperature was determined in 220 patients

^d^Glasgow coma score was determined in 228 patients

^e^CSF leukocyte count was determined in 214 patients

^f^Positive CSF culture was determined in 235 patients

^g^CSF protein was determined in 217 patients

^h^CSF glucose was determined in 202 patients

^i^Positive blood culture was determined in 209 patients

^j^Other is defined as arthritis or dermo-hypodermal infection

### Intracranial complications

**Fig. 1:**
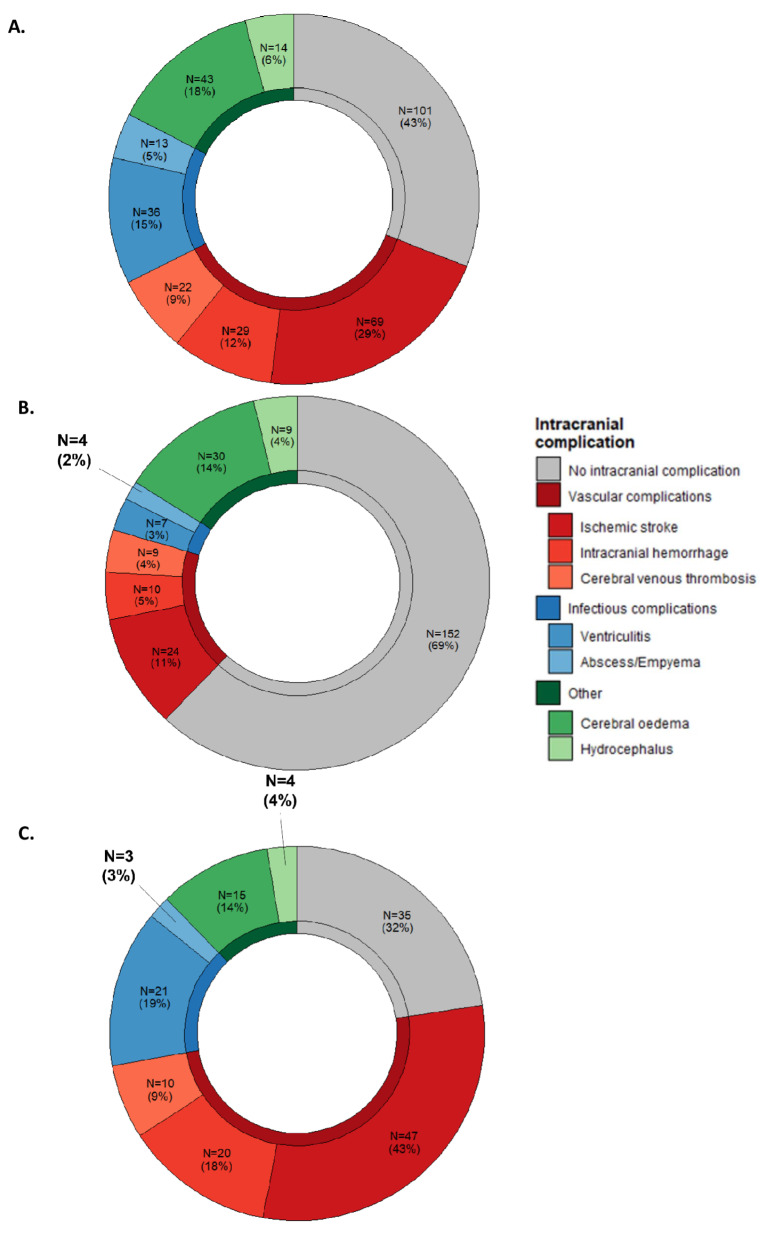
Prevalence of intracranial complications. **A**. Regardless of the time of hospitalization, **B**. On ICU admission, **C**. during ICU stay. *Numbers do not add up to total and percentage to 100% because one patient may have several complications

A total of 383 brain CT scans and 161 brain MRI were performed. In the initial phase of management, the imaging performed was predominantly a brain CT scan in 80% of cases, while MRI became the preferred imaging method in the post-acute phase of care. 124/237 (52%) patients had neuroimaging only at ICU admission, 14/237 (6%) during ICU stay and 96/237 (41%) had neuroimaging at both times. 136/237 patients (57%, 95%CI 0.48–0.66) developed at least one intracranial complication from ICU admission to ICU discharge, with a median of 1 [0–2] intracranial complication per patient. A description of intracranial complication according to neuroimaging type and according to the three time periods is presented in Additional eTable 2 and Fig. 1. Overall, ischemic lesion was the most frequent complication (69/237, 29%), followed by diffuse cerebral oedema (43/237, 18%), ventriculitis (36/237, 15%), intracranial hemorrhage (29/237, 12%), cerebral venous thrombosis (22/237, 9%), hydrocephalus (14/237, 6%) and abscess/empyema (13/237, 6%).

Neuroimaging on ICU admission was available for 220 (93%) patients, with the remaining 17 having their first neuroimaging between 2 and 12 days after ICU admission, of which 68/220 (31%, 95%CI 0.25–0.37) had at least one intracranial complication. At ICU admission, the most frequent intracranial complications were diffuse cerebral oedema (30/220, 14%) and ischemic lesion (24/220, 11%). Intracranial complication at admission was associated with younger patient and focal neurological signs (Additional eTable 3). Presence of a vascular complication at admission was associated with age, signs of shock and focal neurological signs, while diffuse cerebral oedema / hydrocephalus at admission was associated with age and temperature (see Additional eTable 4).

Among patients who underwent neuroimaging during ICU stay, median time between neuroimaging admission and second neuroimaging was 3 days [1–5] and 75/110 patients (68%, 95%CI 0.59–0.77) had at least one intracranial complication. During ICU stay, ischemic lesion was the most frequent (47/110 patients, 43%), followed by ventriculitis (21/110, 19%) and intracranial hemorrhage (20/110, 18%). At ICU discharge, ventriculitis was the most frequent (9/49, 18%), followed by abscess/empyema (6/49, 12%) and ischemic lesion (5/49, 10%). Illustrations of intracranial complications are described in Additional eFigure 2.

### ICU management and hospital outcomes

ICU management and outcomes are described in Additional eTable 5. A total of 170 patients (72%) received mechanical ventilation, for a median duration of 7 days [3–14]. Third generation cephalosporins were the most frequently used empirical antibiotic therapy (231/237, 97%) and 86% (203/237) received adjunctive steroids. Reduced susceptibility to β-lactams was low (15%), and antibiotic de-escalation was made in 58% cases. Amoxicillin was the most frequently used antibiotic treatment after de-escalation, and the median duration of antibiotic therapy was 13 days [9–14]. The duration of ICU and hospital stays were 6 [3–13] days and 16 [10–32] days, respectively. Occurrence of intracranial complications according to time of corticoid withdrawal are showed in Additional eFigure 3.

### Outcomes

At 90 days, 103 patients (44%, 95%CI 37–50%) had unfavorable neurological outcome, including 71 (30%, 95%CI 25–37%) deaths. A total of 64/71 deaths (90%) occurred during ICU stay. A neurological cause of death was identified in 50/64 cases (78%), including 25 brain deaths, 22 withdrawals of care due to poor neurological prognosis, and 3 brain herniations. 50% of patients who died in the intensive care unit had severe brain injury on neuroimaging that led to a decision of life support limitation due to an anticipated poor neurological prognosis. Systemic causes accounted for 12/64 deaths (19%) and two causes of death remained unknown because of hospital transfer. Distribution of scores on the mRS according to intracranial complications are detailed in Additional eFigure 4. The functional outcome assessment by mRS was performed at 90 days [54–99] after ICU admission for patients with favorable outcome and 80 days [39–92] for patients with unfavorable outcome. Patients who had repeated neuroimaging during ICU stay were more severely ill, as reflected by a significantly higher SAPS 2, the number of signs of shock, the lower Glasgow coma score and more unfavorable outcomes compared to the others (see Additional eTable 6).

### Factors associated with unfavorable outcome

**Fig. 2:**
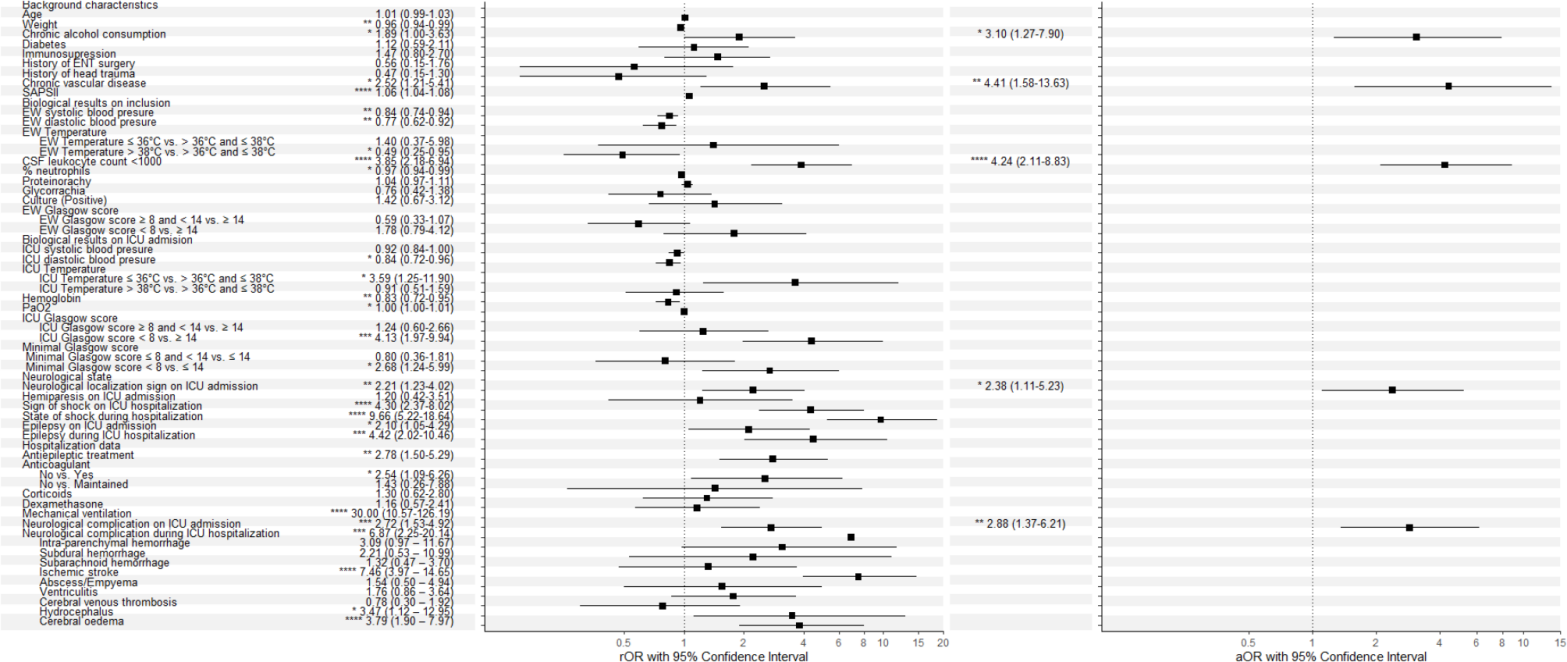
Factors associated with poor outcome identified by logistic regression, multivariate analysis

The non-adjusted associations with unfavorable neurological outcome are detailed in Fig. 2. Among intracranial complications, ischemic lesion (OR 7.46, 95%CI 3.97–14.65), diffuse cerebral oedema (OR 3.79, 95%CI 1.90–7.97) and hydrocephalus (OR 3.37, 95%CI 1.12–12.95) were associated with unfavorable neurological outcome. Based on the univariate logistic regression, the multivariate logistic model was built with 16 variables, with a sample of 196 patients. Other indicators of unfavorable outcome identified in our study included chronic alcohol consumption (aOR 3.10, 95%CI 1.27–7.90), chronic vascular disease (aOR 4.41, 95%CI 1.58–13.63), focal neurological sign (aOR 2.38, 95%CI 1.11–5.23), presence of intracranial complication at ICU admission (aOR 2.88, 95%CI 1.37–6.21) and CSF leukocyte count < 1000 (aOR 4.24, 95%CI 2.11–8.83) (Fig. 2). The distribution of scores on the mRS according to independent predictors of unfavorable outcome is detailed in Additional eFigure 5.

**Fig. 3:**
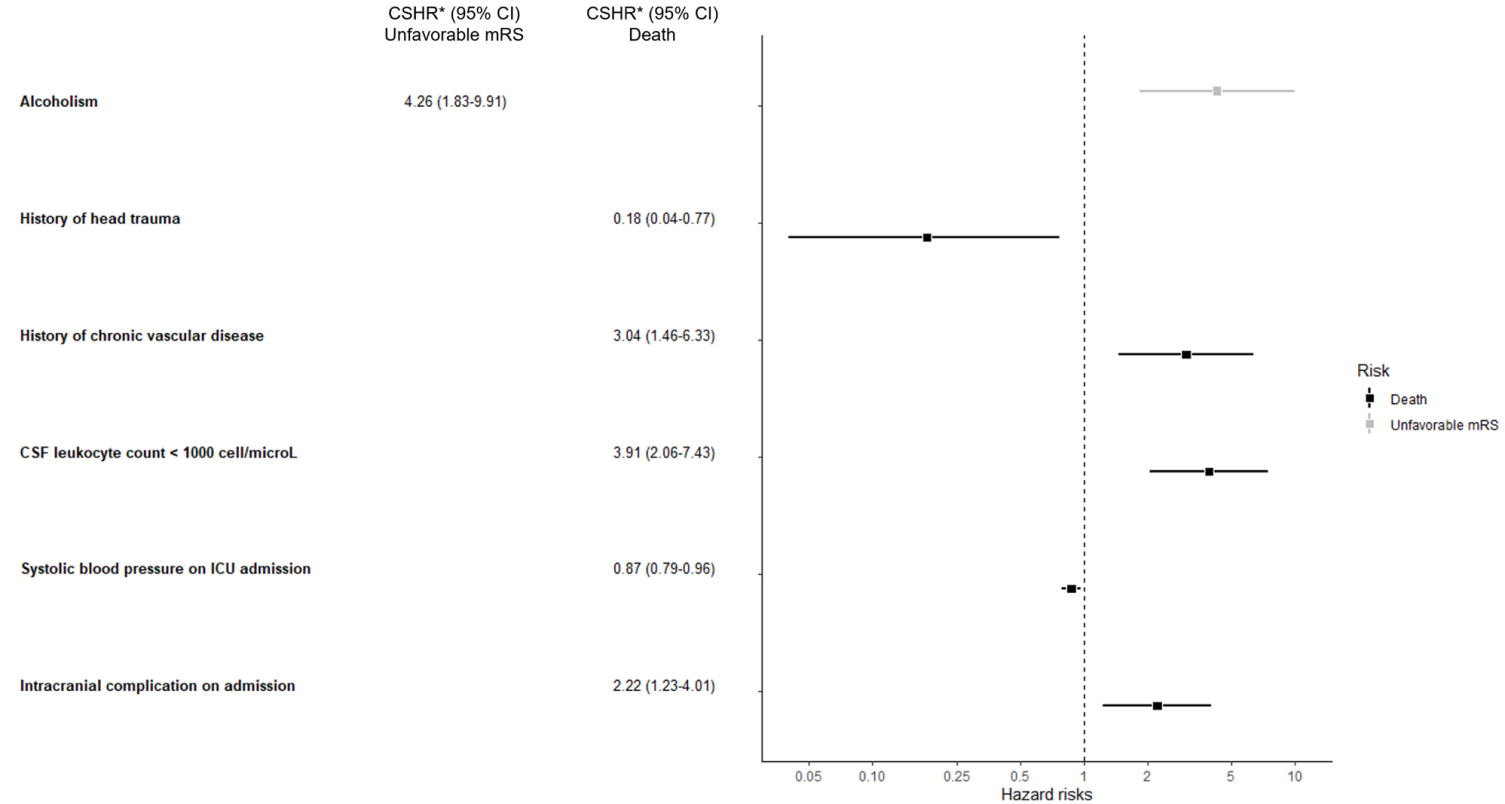
Factors associated with poor outcome identified by multivariate competing risk analysis

**Fig. 4:**
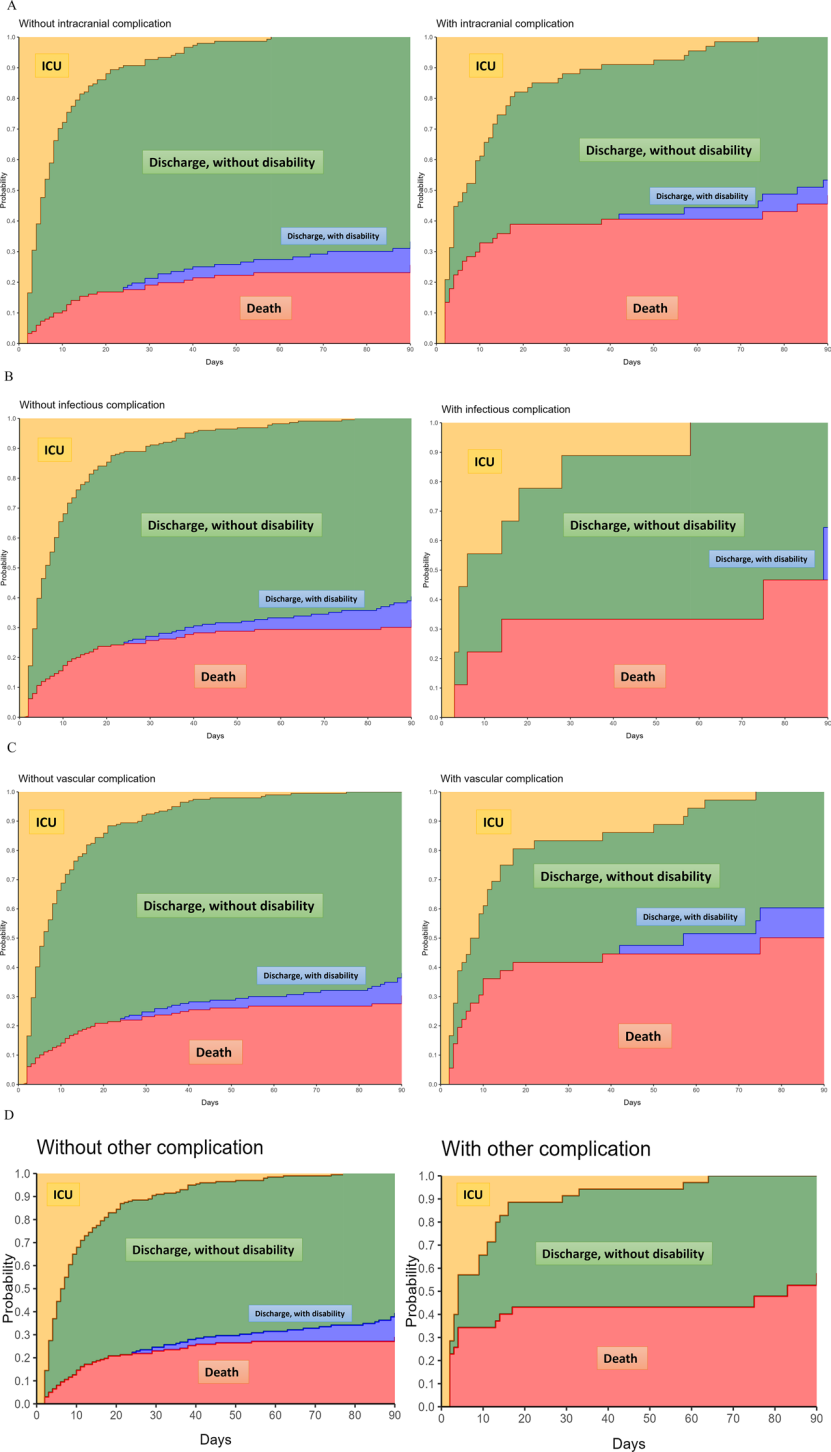
Stacked probability plots. Plots illustrate the state occupation probabilities of being in each state— in ICU, alive and out of ICU without disability, alive and out of ICU with disability or dead—over the 90 days following ICU admission. **A**. For any type of intracranial complications, **B**. For infectious complications, **C**. For vascular complications, **D**. For other intracranial complications

Competing risk analysis, with persistent disability (mRS score 3–5) as the primary risk and ICU-death as the competing risk, revealed that chronic alcohol consumption was the sole significant variable associated with persistent disability at 90 days (Cause-specific Hazard Ratio 4.26, 95%CI 1.83–9.91), whereas the remaining variables were associated with mortality (Fig. 3). The Fine and Gray Sub distribution Hazard model adjusted for illness severity displayed similar results, with a Sub Distribution Hazard Ratio (SDHR) for chronic alcohol consumption of 3.58 (95%CI 1.16-11.00). The cause-specific cumulative hazards of unfavorable functional outcome with death as competing risk according to types of intracranial complication are presented in Fig. 4.

### Outcomes of excluded patients

Post-hoc analyses of the 79 patients staying less than 48 h in ICU showed that 22 of them were extremely ill at admission, as reflected by a median SAPS II of 86 [7094] and died in ICU. The 57 other patients discharged alive from ICU within 48 h had a favorable neurological outcome.

## Discussion

In this multicenter study conducted in adult patients with severe pneumococcal meningitis, we found that intracranial complications, diagnosed on neuroimaging, were detected in 57% of cases during the entire course of the hospitalization (including intracranial complication diagnosed on the first imaging during admission). We identified intracranial complications as detected on neuroimaging as a strong indicator of unfavorable outcome, in the form of death. Most frequent intracranial complications consisted of ischemic lesion and diffuse cerebral oedema. The distribution of intracranial complications differed depending on the time of diagnosis. While cerebrovascular complications and diffuse cerebral edema were highly prevalent at all timepoints, ventriculitis was rarely present on ICU admission, and more prevalent afterwards. In our study, diffuse cerebral edema was most often detected by CT scan studies, while it is well-known that MRI is a more sensitive tool for detecting ischemic lesions. This can be explained by the fact that brain CT scans were performed at an early stage, identifying the most severe patients with increased intracranial pressure or in a state of brain death. MRI was performed later on in survivors, who did not exhibit the most severe clinical characteristics. Considering these results, we can recommend that CT remains a more accessible examination allowing for rapid confirmation of a suspicion of increased intracranial pressure with diffuse cerebral edema or a suspicion of brain death. We suggest that for patients with persistent encephalopathy or remaining unresponsive after sedation interruption, MRI studies should be proposed to detect other intracranial complications which may have important prognostic consequences.

To the best of our knowledge, this is the first study specifically investigating the occurrence of intracranial complications in patients with pneumococcal meningitis throughout the entire duration of the intensive care process. The diagnosis of intracranial complications may dramatically impact on the management of the patient. Of note, the identification of infectious complications (i.e. brain abscess, ventriculitis), may alter the antibiotic regimen and duration of therapy. Other complications may sometimes require additional interventions, such as neurosurgical drainage in case of abscess or empyema, or placement of an external ventricular drainage to manage hydrocephalus. These interventions may need to be performed urgently to prevent further neurological damage. We found very few patients who underwent neurosurgical treatment specifically for treatment of hydrocephalus (*n* = 3) as the number of this complication was small (14 patients). One of the explications was that hydrocephalus was not significant enough to justify external derivation. The diagnosis of intracranial complications may also significantly impact prognostication, in case of severe cerebrovascular complications. Considering our results, a systematic neuroimaging should be considered at least once and repeated in case of unexplained neurological deterioration during ICU stay.

Our study described a representative population of severely affected patients with high compliance to international guidelines [17]. We observed the use of empirical 3rd generation cephalosporin antibiotic therapy and adjunctive steroids in 97% and 86% of cases, respectively. In contrast, a recent retrospective cohort study in the UK and Ireland showed that only 57% patients with pneumococcal meningitis received steroids [30]. We did not observe any association between steroid administration and intracranial complications. Of note, corticosteroid withdrawal at day 4 was not associated with new intracranial complications, notably vascular events.

Previous studies evaluated the occurrence of intracranial complications in patients with pneumococcal meningitis. A nationwide multicenter study identified a high number of intracranial complications at the acute phase of the disease, mainly ischemic lesion and diffuse cerebral edema [9]. Another study conducted in the ICU setting described lower frequencies of such complications, as compared to our study [31, 32]. The higher incidence of intracranial complications observed in our study can potentially be attributed to several factors. First, the evaluation of these complications was conducted at various timepoints, allowing for a comprehensive assessment of their occurrence. Additionally, a wider range of complications was included in our analysis, leading to a more extensive categorization.

Other indicators of unfavorable outcome identified in our study included chronic alcohol consumption, chronic vascular disease, focal neurological sign, and CSF leukocyte count < 1000 cell/microL at admission, as previously described [2, 10]. The association between chronic vascular disease and poor neurological outcome is likely linked to patients’ cardiovascular comorbidities. Using competing risk analyses, we identified chronic alcohol consumption as the only parameter associated with persistent disability at 90 days in survivors, while intracranial complication at ICU admission was associated with death. Several studies already identified chronic alcohol consumption as a poor outcome variable [2, 33, 34]. Chronic alcohol consumption has been linked to increased host susceptibility to bacterial pneumonia due to alteration of immune regulation leading to immunodeficiency [35]. In a nationwide observational cohort study including 696 episodes of bacterial meningitis occurring in 671 patients, alcoholics were more linked to pneumococcal meningitis and were at risk for unfavorable outcome compared to non-alcoholic patients [36].

Our study has several strengths, including a representative population of severely affected patients. All neuroimaging were reinterpreted by an independent neurologist who was blinded to the patients’ outcome and radiologist’s report with an extensive description of intracranial complications. We used competing risk analyses to identify relevant ICU admission or ICU stay factors associated with unfavorable outcome, and we adjusted analyses for non-neurologic organ failure.

Our study has some limitations, inherent in its retrospective design. First, the time of primary endpoint evaluation was heterogeneous, as only 39% of patients discharged alive had an accurate evaluation of functional status on mRS at 90 days (+/-15 days). Of note, most patients evaluated before 90 days had favorable neurological outcomes. Second, we excluded patients with ICU stays < 48 h, as these patients did not represent a relevant population for neuroprognostication in ICU. Indeed, most of short stayers were discharged alive with favorable neurological outcomes at 90 days, and the remainders had a fulminant course and died within 48 h after ICU admission. Third, neither the timing nor the technique of neuroimaging was standardized between centers. The accurate evaluation of intracranial complications on the day of ICU admission was impossible if there was no imaging performed at this time, particularly for ischemic lesion as the distinction between acute and sequels can be relatively difficult if only a CT scan was performed. Thus, immortality bias may have been introduced with complications on admission being missed, leading to classification bias. Nevertheless, neuroimaging is typically initiated in response to neurological decline (e.g., presence of focal neurological signs, occurrence of epilepsy), which can be inferred as the approximate occurrence time of intracranial complications. Among patients alive at ICU discharge, underestimation of intracranial complications may have been less important, as it is more likely that physicians do not require brain imaging if they do not suspect an intracranial complication.

## Conclusion

In severely affected adult patients with pneumococcal meningitis, intracranial complications diagnosed on neuroimaging at admission or during ICU stay were observed in 57% of cases. Intracranial complications were independently associated with a higher risk of poor functional outcome, in the form of persistent disability or death. This study highlights the diagnostic and prognostic value of neuroimaging studies performed in this population.

## Electronic supplementary material

Below is the link to the electronic supplementary material.

Supplementary Material 1

## Data Availability

The datasets used and/or analyzed during the current study are available from the corresponding author on reasonable request.
