# Supplementary material for: Intracranial complications in adult patients with severe pneumococcal meningitis: a retrospective multicenter cohort study
Source: Ann Intensive Care. 2024 Dec 19;14:182. doi: 10.1186/s13613-024-01405-z (PMC11659536; doi:10.1186/s13613-024-01405-z)
Supplement: Supplementary file 1 — Supplementary Material 1 [file 13613_2024_1405_MOESM1_ESM.docx]

Online-Only Supplements and Multimedia

[eTable 1 - Radiological definitions of intracranial complication 2](#_Toc177373976)

[eFigure 1- Flow chart 5](#_Toc177373977)

[eTable 2 - Intracranial complications according to image type 6](#_Toc177373978)

[eTable 3 - Patients’ characteristics according to the presence of intracranial complication at admission 7](#_Toc177373979)

[eTable 4 - Patients’ characteristics according to the presence of vascular, infectious and other* intracranial complication at admission 8](#_Toc177373980)

[eFigure 2 – Illustrations of intracranial complications 10](#_Toc177373981)

[eTable 5 – ICU management and hospital outcomes 11](#_Toc177373982)

[eTable 6 – Comparison of baseline characteristics and outcomes according to the number of neuroimaging per patient 12](#_Toc177373983)

[eFigure 3 - Occurrence of intracranial complications according to time of corticoid withdrawal 13](#_Toc177373984)

[eFigure 4 – Distribution of modified Rankin scale according to intracranial complications among 236 patients * 14](#_Toc177373985)

[eFigure 5 - Distribution of modified Rankin scale according to multivariate predictors of unfavorable neurological outcome on 236 patients. 15](#_Toc177373986)

**eTable 1** - Radiological definitions of intracranial complication

| **Intracranial complication** | **CT** | **MRI** | **References** |
| --- | --- | --- | --- |
| **Ischemic lesion** | **NoncontrastCT:**  (a) subtle hypoattenuation, (b) obscuration and loss of gray matter–white matter differentiation in the basal ganglia  (c) cortical sulcal effacement,  (d) loss of the insular ribbon, (e) hyperattenuation of a large vessel (“hyper-attenuating MCA sign” or “dot sign” in an M2 branch)  Use of Alberta Stroke Program Early CT Score  **CT angiography:** arterial obstruction | Hyperintense on DW images and hypointense on ADC images at the acute phase.  Hyperintense lesions on T2-weighted and fluid-attenuated inversion recovery MR images after a few hours (classically > 6hours) | Wardlaw JM, Mielke O. Early signs of brain infarction at CT: observer reliability and outcome after thrombolytic treatment—systematic review. *Radiology 2005;235:444–453*  Barber PA, Demchuk AM, Zhang J, Buchan AM. Validity and reliability of a quantitative computed tomography score in predicting outcome of hyperacute stroke before thrombolytic therapy. ASPECTS Study Group. Alberta Stroke Programme Early CT Score. *Lancet 2000;355:1670–1674* |
| **Diffuse cerebral oedema** | Disappearance of sulcal relief  Completely effaced basal cisterns  Obscuration and loss of gray matter–white matter differentiation | Disappearance of sulcal relief  Completely effaced basal cisterns | Stefanie Lietke, Stefan Zausinger, Maximilian Patzig, Markus Holtmanspötter, Mathias Kunz.CT-Based Classification of Acute Cerebral Edema: Association with Intracranial Pressure and Outcome. J *Neuroimaging. 2020* Sep;30(5):640-647. |
| **Acute hydrocephalus** | **NoncontrastCT:** Rounding of the frontal horns of the lateral ventricles and dilatation of the temporal horns. Bulging of the floor and the lateral walls of the third ventricle can be seen. | Dilatation of the frontal ventricles, third ventricle or temporal horns. Periventricular T2-weighted and fluid-attenuated inversion recovery hyperintensities from transependymal flow. | Maller VV, Gray RI. Noncommunicating Hydrocephalus. *Semin Ultrasound CT MR. 2016 Apr;37(2):109-19.* |
| **Cerebral venous thrombosis** | **NoncontrastCT:**  Hyperdensity of a cortical vein or dural sinus. Thrombosis of the posterior portion of the superior sagittal sinus: dense or filled delta sign.  **Contrast-enhanced CT**:  Enhancement of the dural lining of the sinus with a filling defect within the vein or sinus.  Classic “empty delta” sign: a central hypodensity due to very slow or absent flow within the sinus is surrounded by contrast enhancement in the surrounding triangular shape in the posterior aspect of the superior sagittal sinus. | **NoncontrastMRI:**  Absence of a fluid void signal in the sinus, T2 hypointensity suggestive of a thrombus  **Contrast-enhanced MRI:** central Isodense lesion in a venous sinus with surrounding enhancement | Leach JL, Fortuna RB, Jones BV, Gaskill-Shipley MF. Imaging of cerebral venous thrombosis: current techniques, spectrum of findings, and diagnostic pitfalls. *Radiographics. 2006;26(suppl 1):S19–S41.*  Ford K, Sarwar M. Computed tomography of dural sinus thrombosis. *AJNR Am J Neuroradiol. 1981;2:539–543*  Bianchi D, Maeder P, Bogousslavsky J, Schnyder P, Meuli RA. Diagnosis of cerebral venous thrombosis with routine magnetic resonance: an update*. Eur Neurol. 1998;40:179–190* |
| **Ventriculitis** | **Contrast-enhanced CT**: Ependymal enhancement | Ependymal thickening and enhancement with T2 prolongation surrounding the ventricles are often seen  Hyperintense on DW images and hypointense on ADC images | Saman Hazany, John L Go, Meng Law. Magnetic resonance imaging of infectious meningitis and ventriculitis in adults. *Top Magn Reson Imaging. 2014 Oct;23(5):315-25.* |
| **Abcess** | **Contrast-enhanced CT**: Enhancing capsular ring, surrounding hypodensity edema | Fluid intensity collection at the gray-white matter junction, with decreased diffusion; surrounding vasogenic edema; and well-defined, smooth, enhancing capsular ring, which is markedly hypointense on T2W MR images. | Saman Hazany, John L Go, Meng Law. Magnetic resonance imaging of infectious meningitis and ventriculitis in adults. *Top Magn Reson Imaging. 2014 Oct;23(5):315-25.* |
| **Empyema** | Hypodense pericerebral or interhemispheric collection with a density slightly higher than that of CSF; contrast enhancement is noted on the wall of the collection. | Hypointense on T1 with a signal slightly more intense than that of CSF and hyperintense on T2, with a signal close to that of CSF; on FLAIR, the signal is hyperintense; intense contrast enhancement is noted at the periphery of the lesion. Hyperintense signal related to a decrease in ADC. | Ramsay DW, Aslam M, Cherryman GR. Diffusion-weighted imaging of cerebral abscess and and subdural empyema. AJNR Am J Neuroradiol 2000 ; 21 : 1172.  Tsuchiya K, Osawa A, Katase S et al. Diffusion-weighted MRI of subdural and epidural empyemas. Neuroradiology 2003 ; 45 : 220–3.  Tsuchiya K, Makita K, Furui S et al. Contrast-enhanced magnetic resonance imaging of sub-and epidural empyemas. Neuroradiology 1992 ; 34 : 494–6. |

# eFigure 1- Flow chart


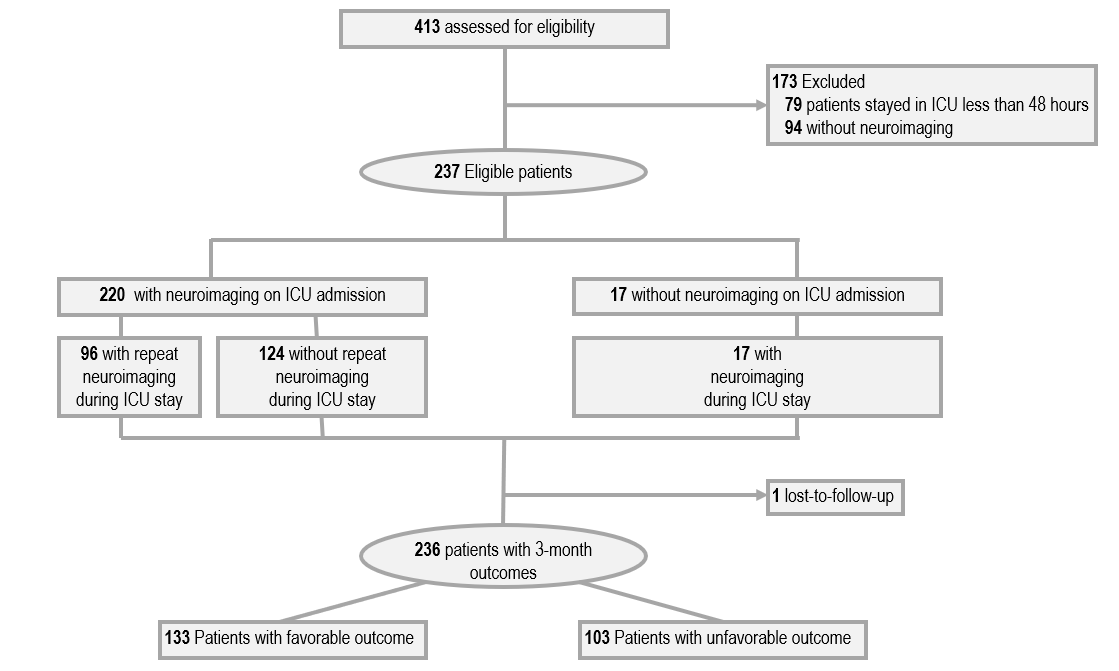


# eTable 2 - Intracranial complications according to image type

|  | **CT**  **N = 128** | **MRI**  **N = 147** |
| --- | --- | --- |
| Cerebral Oedema | 45 (35) | 2 (1) |
| Ischemic stroke | 26 (20) | 57 (39) |
| Hydrocephalus | 13 (10) | 2 (1) |
| Subarachnoid hematoma | 9 (7) | 8 (5) |
| Intraparenchymal hematoma | 9 (7) | 6 (4) |
| Cerebral venous thrombosis | 8 (6) | 16 (11) |
| Ventriculitis | 5 (4) | 27 (18) |
| Subdural hematoma | 5 (4) | 4 (3) |
| Abscess/Empyema | 2 (2) | 13 (9) |

Data are number/N (%).

# eTable 3 - Patients’ characteristics according to the presence of intracranial complication at admission

|  | **All patients**  **N = 237** | **No intracranial complication at admission**  **N = 151** | **Intracranial complication at admission**  **N = 68** | ***P Value*** |
| --- | --- | --- | --- | --- |
| **Demographics** |  |  |  |  |
| Age (years) | 60 [49 - 69] | 62 [52 - 72] | 52 [45 - 64] | **<0.001** |
| Male sex | 129 (54) | 83 (55) | 37 (54) | >0.99 |
| **Medical history** |  |  |  |  |
| Diabetes | 48 (20) | 32 (21) | 12 (18) | 0.67 |
| Chronic alcohol consumption | 48 (20) | 30 (20) | 14 (21) | >0.99 |
| Immunosuppression | 57 (24) | 33 (22) | 19 (28) | 0.42 |
| Head trauma | 18 (8) | 14 (9) | 4 (6) | 0.56 |
| Recent NSAID use | 36 (15) | 22 (15) | 11 (16) | 0.92 |
| Chronic vascular disease | 35 (15) | 27/150 (18) | 5/67 (8) | 0.069 |
| **Clinical presentation** |  |  |  |  |
| SAPS 2 | 42 [31 - 58] | 41 [30 - 56] | 44 [34 - 60] | 0.12 |
| Signs of shock | 68/237 (29) | 37 (25) | 23 (34) | 0.21 |
| Temperature (°C)^a^ |  |  |  | 0.59 |
| ≤ 36°C | 18/220 (8) | 10/142 (7) | 7/63 (11) |  |
| > 36°C and ≤ 38°C | 101/220 (46) | 65/142 (46) | 29/63 (46) |  |
| > 38°C | 101/220 (46) | 67/142 (47) | 27/63 (43) |  |
| Glasgow coma score^b^ |  |  |  | 0.24 |
| ≥ 14 | 45/228 (20) | 28/145 (19) | 9/66 (14) |  |
| ≥ 8 and < 14 | 115/228 (50) | 78/145 (54) | 32/66 (48) |  |
| < 8 | 68/228 (30) | 39/145 (27) | 25/66 (38) |  |
| Focal neurological signs | 63 (27) | 35 (23) | 27 (40) | **0.019** |
| Seizures | 39 (16) | 26 (17) | 12 (18) | >0.99 |
| **Blood and CSF findings** |  |  |  |  |
| CSF leukocyte count^c^ |  |  |  |  |
| cell / microL | 970 [101 – 3500] | 1,000 [107 - 3,500] | 800 [105 - 3,800] | 0.93 |
| <1000 cell/microL | 108/214 (46) | 67/137 (49) | 34/63 (54) | 0.61 |
| % of neutrophils | 92 [85 – 96] | 92 [86 - 96] | 92 [84 - 97] | 0.96 |
| Positive CSF Culture^d^ | 202/235 (86) | 129/150 (86) | 60 (88) | 0.81 |
| CSF protein (g/l)^e^ | 4.9 [3.0 – 7.2] | 4.6 [3.0 - 7.0] | 5.6 [3.4 - 8.6] | 0.077 |
| CSF glucose < 2.5 mmol/l^f^ | 156/202 (77) | 95/128 (74) | 49/61 (80) | 0.46 |
| Positive blood culture^g^ | 126/209 (60) | 83/135 (61) | 33/60 (55) | 0.49 |

Data are median [Interquartile Range] or number/N (%).

*mRS* modified Rankin Scale; *SAPS II* Simplified Acute Physiology Score; *CSF* cerebrospinal fluid; *NSAID* nonsteroidal anti-inflammatory drugs

^a^Temperature was determined in 220 patients

^b^ Glasgow coma score was determined in 228 patients

^c^ CSF leukocyte count was determined in 214 patients

^d^ Positive CSF culture was determined in 235 patients

^e^ CSF protein was determined in 217 patients

^f^ CSF glucose was determined in 202 patients

^g^ Positive blood culture was determined in 209 patients

# eTable 4 - Patients’ characteristics according to the presence of vascular, infectious and other* intracranial complication at admission

|  | **All patients**  **N = 237** | **No Vascular complication at admission**  **N = 199** | **Vascular complication at admission**  **N = 37** | ***P Value*** | **No Infectious complication at admission**  **N = 227** | **Infectious complication at admission**  **N = 9** | ***P Value*** | **No Other intracranial complication at admission**  **N = 201** | **Other intracranial complication at admission**  **N = 35** | ***P Value*** |
| --- | --- | --- | --- | --- | --- | --- | --- | --- | --- | --- |
| **Demographics** |  |  |  |  |  |  |  |  |  |  |
| Age (years) | 60 [49 - 69] | 62 [50 - 71] | 52 [46 - 62] | **0.011** | 60 [49 - 69] | 64 [52 - 70] | 0.72 | 62 [50 - 71] | 51 [38 - 58] | **<0.001** |
| Male sex | 129 (54) | 108 (54) | 21 (57) | 0.92 | 124 (55) | 5 (56) | >0.99 | 112 (56) | 17 (49) | 0.55 |
| **Medical history** |  |  |  |  |  |  |  |  |  |  |
| Diabetes | 48 (20) | 42 (21) | 6 (16) | 0.65 | 46 (20) | 2 (22) | >0.99 | 44 (22) | 4 (11) | 0.23 |
| Chronic alcohol consumption | 48 (20) | 39 (20) | 9 (24) | 0.66 | 46 (20) | 2 (22) | >0.99 | 39 (19) | 9 (26) | 0.53 |
| Immunosuppression | 57 (24) | 45 (23) | 9 (24) | 0.99 | 53 (23) | 1 (11) | 0.65 | 42 (21) | 12 (34) | 0.13 |
| Head trauma | 18 (8) | 18 (9) | 0 (0) | 0.12 | 18 (8) | 0 (0) | 0.81 | 15 (8) | 3 (9) | >0.99 |
| Recent NSAID use | 36 (15) | 31 (16) | 5 (14) | 0.94 | 36 (16) | 0 (0) | 0.41 | 28 (14) | 8 (23) | 0.27 |
| Chronic vascular disease | 35/235 (15) | 32/197 (16) | 3 (8) | 0.31 | 35/226 (15) | 0/8 (0) | 0.48 | 34 (17) | 1 (3) | 0.055 |
| **Clinical presentation** |  |  |  |  |  |  |  |  |  |  |
| SAPS 2 | 42 [31 - 58] | 42 [31 - 57] | 42 [34 - 61] | 0.42 | 42 [31 - 58] | 37 [30 - 61] | 0.68 | 41 [30 - 56] | 47 [39 - 59] | 0.073 |
| Signs of shock | 68 (29) | 51 (26) | 16 (43) | **0.047** | 67 (30) | 0 (0) | 0.12 | 56 (28) | 11 (31) | 0.82 |
| Temperature (°C)^a^ |  |  |  | 0.65 |  |  | 0.38 |  |  | **0.008** |
| ≤ 36°C | 18/220 (8) | 16/185 (9) | 2/34 (6) |  | 18/210 (9) | 0 (0) |  | 12/187 (6) | 6/32 (19) |  |
| > 36°C and ≤ 38°C | 101/220 (46) | 86/185 (46) | 14/34 (41) |  | 97/210 (46) | 3 (33) |  | 82/187 (44) | 18/32 (56) |  |
| > 38°C | 101/220 (46) | 83/185 (45) | 18/34 (53) |  | 95/210 (45) | 6 (67) |  | 93/187 (50) | 8/32 (25) |  |
| Glasgow coma score^b^ |  |  |  | 0.11 |  |  | 0.87 |  |  | 0.23 |
| ≥ 14 | 45/228 (20) | 39/191 (20) | 5/36 (14) |  | 42/218 (19) | 2 (22) |  | 40/193 (21) | 4/34 (12) |  |
| ≥ 8 and < 14 | 115/228 (50) | 100/191 (52) | 15/36 (42) |  | 110/218 (50) | 5 (56) |  | 99/193 (51) | 16/34 (47) |  |
| < 8 | 68/228 (30) | 52/191 (27) | 16/36 (44) |  | 66/218 (30) | 2 (22) |  | 54/193 (28) | 14/34 (41) |  |
| Focal neurological signs | 63 (27) | 46/199 (23) | 17 (46) | **0.007** | 59 (26) | 4 (44) | 0.40 | 49 (24) | 14 (40) | 0.085 |
| Seizures | 39 (16) | 35/199 (18) | 4 (11) | 0.44 | 39 (17) | 0 (0) | 0.37 | 32 (16) | 7 (20) | 0.72 |
| **Blood and CSF findings** |  |  |  |  |  |  |  |  |  |  |
| CSF leukocyte count^c^ |  |  |  |  |  |  |  |  |  |  |
| cell / microL | 970 [101 – 3500] | 1000 [98 - 3500] | 795 [225 - 2875] | 0.95 | 995 [104 - 3500] | 238 [93 - 2300] | 0.85 | 1000 [100 - 3400] | 485 [108 - 6200] | 0.91 |
| <1000 cell/microL | 108/214 (46) | 89/179 (50) | 19/34 (56) | 0.64 | 102/204 (50) | 6 (67) | 0.52 | 90/181 (50) | 18/32 (56) | 0.62 |
| % of neutrophils | 92 [85 – 96] | 93 [86 - 97] | 89 [77 - 95] | 0.074 | 92 [86 - 96] | 92 [84 - 97] | 0.92 | 92 [85 - 96] | 95 [88 - 99] | 0.14 |
| Positive CSF Culture^d^ | 202/235 (86) | 166/197 (84) | 36 (97) | 0.063 | 195/225 (87) | 7 (78) | 0.79 | 172/199 (86) | 30 (86) | >0.99 |
| CSF protein (g/l)^e^ | 4.9 [3.0 – 7.2] | 4.7 [3.0 - 7.1] | 6.1 [3.9 - 7.6] | 0.069 | 4.8 [3.0 - 7.2] | 6.9 [6.0 - 11.0] | 0.089 | 4.8 [3.0 - 7.2] | 5.1 [3.5 - 9.2] | 0.26 |
| CSF glucose < 2.5 mmol/l^f^ | 156/202 (77) | 128/168 (76) | 28/34 (82) | 0.58 | 147/193 (76) | 9 (100) | 0.21 | 132/172 (77) | 24/30 (80) | 0.88 |
| Positive blood culture^g^ | 126/209 (60) | 62/117 (53) | 64/92 (70) | **0.022** | 120/200 (60) | 6 (67) | 0.96 | 110/179 (61) | 16/30 (53) | 0.52 |

Data are median [Interquartile Range] or number/N (%).

*mRS* modified Rankin Scale; *SAPS II* Simplified Acute Physiology Score; *CSF* cerebrospinal fluid; *NSAID* nonsteroidal anti-inflammatory drugs

^a^Temperature was determined in 220 patients

^b^ Glasgow coma score was determined in 228 patients

^c^ CSF leukocyte count was determined in 214 patients

^d^ Positive CSF culture was determined in 235 patients

^e^ CSF protein was determined in 217 patients

^f^ CSF glucose was determined in 202 patients

^g^ Positive blood culture was determined in 209 patients

*Other complications regrouped oedema and hydrocephalus

# eFigure 2 – Illustrations of intracranial complications

B

A


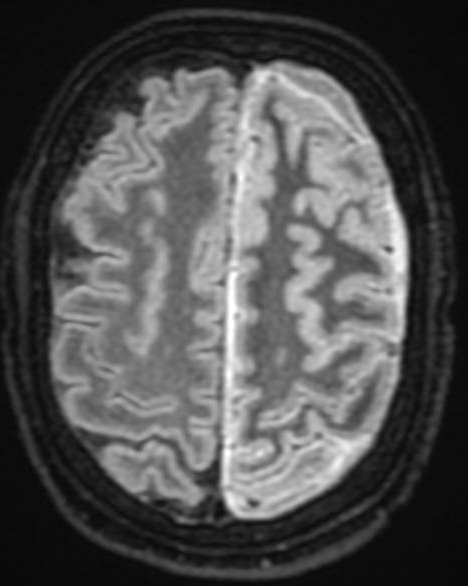

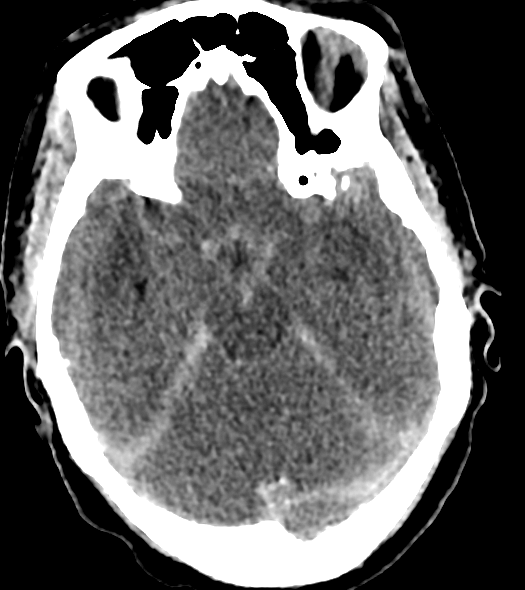

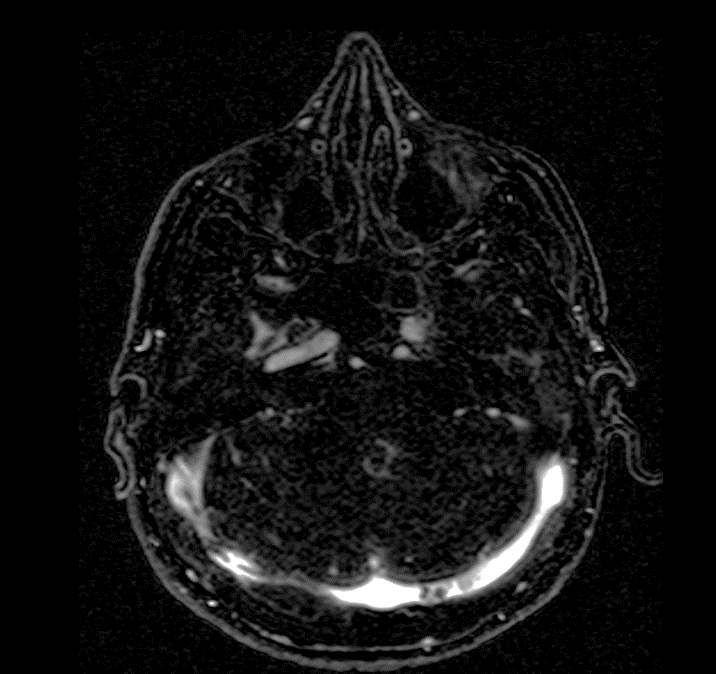

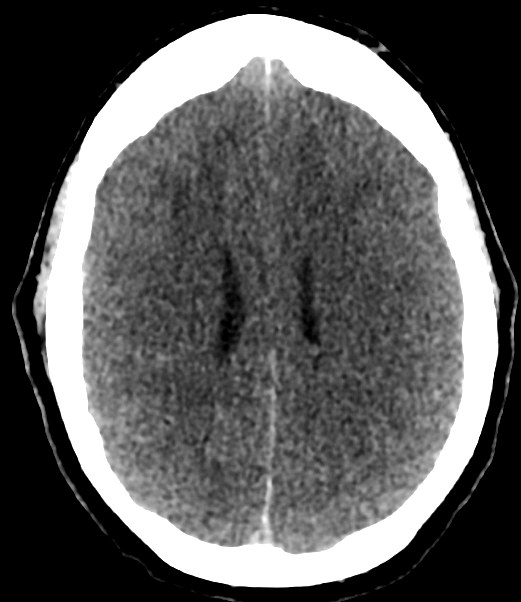

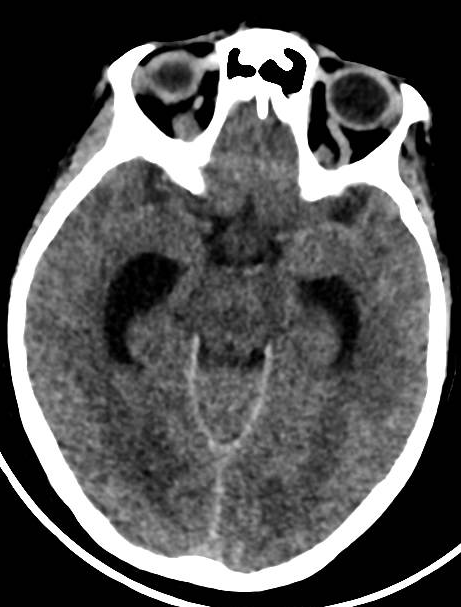

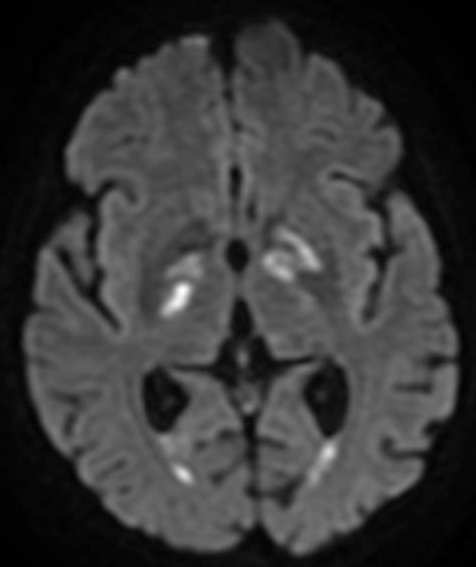


F

E

D

C

**A)** Empyema. **B)** Intracranial Hemorrhage. **C)** Cerebral venous thrombosis. **D)** Cerebral oedema. **E)** Hydrocephalus.

**F)** Ischemic stroke and ventriculitis

# eTable 5 – ICU management and hospital outcomes

|  | **All patients**  **N=237** | **mRS 0-2**  **N = 133** | **mRS 3-6**  **N = 103** | **P Value** |
| --- | --- | --- | --- | --- |
| ICU management |  |  |  |  |
| Empirical 3^rd^ generation cephalosporin | 231 (97) | 130 (98) | 100 (97) | >0.99 |
| Adjunctive steroids | 203 (86) | 112 (84) | 90 (87) | 0.49 |
| Mechanical ventilation | 170 (72) | 70 (53) | 100 (97) | **<0.01** |
| Antiepileptic drugs | 54 (23) | 20 (15) | 34 (33) | **0.01** |
| Anticoagulation^a^ |  |  |  | 0.09 |
| No | 204/236 (86) | 120/132 (91) | 84/103 (82) |  |
| Yes | 26/236 (11) | 9/132 (7) | 16/103 (16) |  |
| Maintained | 6/236 (3) | 3/132 (2) | 3/103 (3) |  |
| Anti-platelet drugs |  |  |  | 0.58 |
| No | 223 (94) | 127 (95) | 95 (91) |  |
| Yes | 7 (3) | 3 (2) | 4 (4) |  |
| Maintained | 7 (3) | 3 (2) | 4 (4) |  |
| ICU and hospital outcomes |  |  |  |  |
| Shock | 80 (34) | 18 (14) | 62 (60) | **<0.01** |
| Seizures during ICU hospitalization | 34 (14) | 9 (7) | 25 (24) | **<0.01** |
| Length of mechanical ventilation, days | 7 [3-14] | 5 [3 – 8] | 9.5 [4 – 17] | **<0.01** |
| Length of stay in ICU, days | 6 [3 – 13] | 4 [3 – 8] | 11 [4 – 21] | **<0.01** |
| Length of stay in hospital, days | 16 [10 – 32] | 17 [14 – 32] | 12 [5 – 32] | **<0.01** |

Data are median [Interquartile Range] or n/N (%). *ICU* Intensive Care Unit

^a^ Anticoagulation was determined in 236 patients

# eTable 6 – Comparison of baseline characteristics and outcomes according to the number of neuroimaging per patient

|  | **All patients**  **N = 237** | **One neuroimaging**  **N = 141** | **> 1 neuroimaging**  **N = 96** | ***P* Value** |
| --- | --- | --- | --- | --- |
| **Demographics** |  |  |  |  |
| Age (years) | 60 [49 - 69] | 60 [48 - 70] | 61 [51 - 67] | 0.80 |
| Male sex | 129 (54) | 72 (51) | 57 (59) | 0.21 |
| **Medical history** |  |  |  |  |
| Diabetes | 48 (20) | 32 (23) | 16 (17) | 0.26 |
| Chronic alcohol consumption | 48 (20) | 26 (18) | 22 (23) | 0.40 |
| Immunosuppression | 57 (24) | 35 (25) | 20 (21) | 0.48 |
| Head trauma | 18 (8) | 10 (7) | 8 (8) | 0.72 |
| Recent NSAID use | 36 (15) | 20 (14) | 16 (17) | 0.60 |
| Chronic vascular disease | 11/235 (5) | 8/140 (6) | 3/95 (3) | 0.53 |
| **Clinical presentation at admission** |  |  |  |  |
| SAPS 2 | 42 [31 - 58] | 38 [28 – 54] | 48 [34 – 61] | **<0.01** |
| Systolic blood pressure^a^ (mmHg) | 130 [110 – 155] | 130 [113 – 156] | 132 [109 – 152] | 0.66 |
| Signs of shock | 68 (29) | 33 (23) | 35 (36) | **0.029** |
| Temperature^c^ (°C) |  |  |  | 0.91 |
| ≤ 36°C | 18/220 (8) | 10/130 (8) | 8/90 (9) |  |
| > 36°C and ≤ 38°C | 101/220 (46) | 61/130 (47) | 40/90 (44) |  |
| > 38°C | 101/220 (46) | 59/130 (45) | 42/90 (47) |  |
| Glasgow coma score^d^ |  |  |  | **0.032** |
| ≥ 14 | 45/228 (20) | 32/134 (24) | 13/94 (14) |  |
| ≥ 8 and < 14 | 115/228 (50) | 70/134 (52) | 45/94 (48) |  |
| < 8 | 68/228 (30) | 32/134 (24) | 36/94 (38) |  |
| Focal neurological signs | 63 (27) | 33 (23) | 30 (31) | 0.18 |
| Seizures | 39 (16) | 16 (12) | 23 (22) | **0.017** |
| **Blood and CSF findings** |  |  |  |  |
| CSF leukocyte count |  |  |  |  |
| cell / microL^e^ | 970 [101 – 3500] | 1190 [175 – 5025] | 482 [90 – 2425] | **0.068** |
| <1000 cell/microL^e^ | 108/214 (46) | 57/126 (45) | 51/88 (58) | **0.067** |
| % of neutrophils^f^ | 92 [85 – 96] | 92 [86 – 96] | 92 [84 – 98] | 0.96 |
| Positive CSF Culture^g^ | 202/235 (86) | 116/139 (83) | 86/96 (90) | 0.18 |
| CSF protein^h^ (g/l) | 4.9 [3.0 – 7.2] | 5.0 [3.0 – 7.2] | 4.8 [3.0 – 7.1] | 0.94 |
| CSF glucose < 2.5 mmol/l^i^ | 156/202 (77) | 91/118 (77) | 65/84 (77) | 0.97 |
| Positive blood culture^j^ | 126/209 (60) | 69/123(56) | 57/86 (66) | 0.30 |
| **Causes of meningitis** |  |  |  | **<0.01** |
| Pneumonia | 41 (17) | 25 (18) | 16 (17) |  |
| Otitis or sinusitis | 98 (41) | 59 (42) | 39 (41) |  |
| Cerebrospinal fluid leak | 12 (5) | 8 (6) | 4 (4) |  |
| Endocarditis | 7 (3) | 2 (1) | 5 (5) |  |
| Other^k^ | 6 (3) | 4 (3) | 2 (2) |  |
| Unknown | 73 (31) | 43 (30) | 30 (31) |  |
| **Neurological and functional outcome** |  |  |  |  |
| mRS > 2 at D90 | 103/236 (44) | 41/140 (29) | 62 (65) | **<0.001** |

Data are median [Interquartile Range] or number/N (%).

*mRS* modified Rankin Scale; *SAPS II* Simplified Acute Physiology Score; *CSF* cerebrospinal fluid; *NSAID* nonsteroidal anti-inflammatory drugs

^a^ Systolic blood pressure was determined in 214 patients ^c^ Temperature was determined in 220 patients ^d^ Glasgow coma score was determined in 228 patients ^e^ CSF leukocyte count was determined in 214 patients ^f^ % of neutrophils was determined on 167 patients ^g^ Positive CSF culture was determined in 235 patients ^h^ CSF protein was determined in 217 patients ^i^ CSF glucose was determined in 202 patients ^j^ Positive blood culture was determined in 209 patients ^k^ Other is defined as arthritis or dermo-hypodermal infection

# eFigure 3 - Occurrence of intracranial complications according to time of corticoid withdrawal


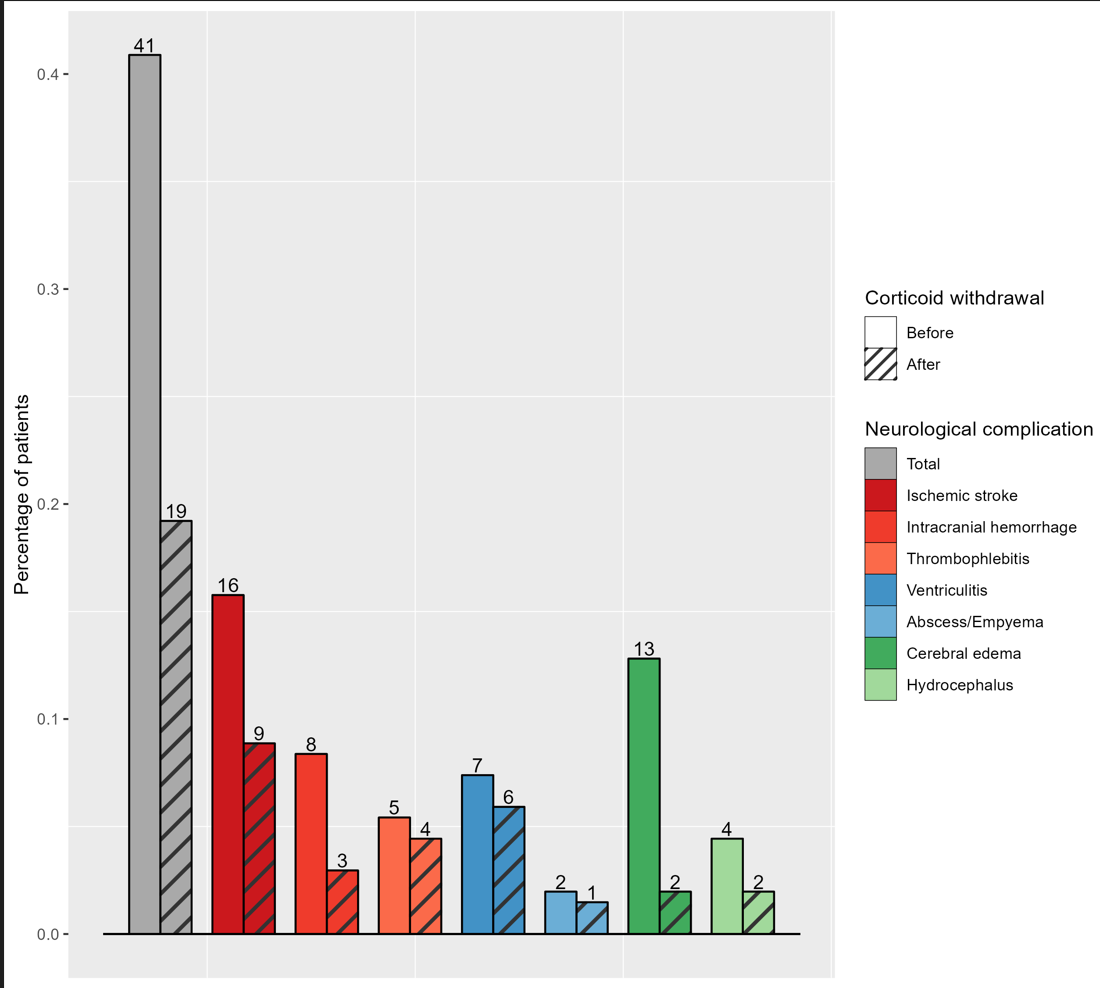


Total

Ischemic stroke

Intracranial hemorrhage

Cerebral venous thrombosis

Ventriculitis

Abscess/Empyema

Cerebral oedema

Hydrocephalus

Intracranial complication

#
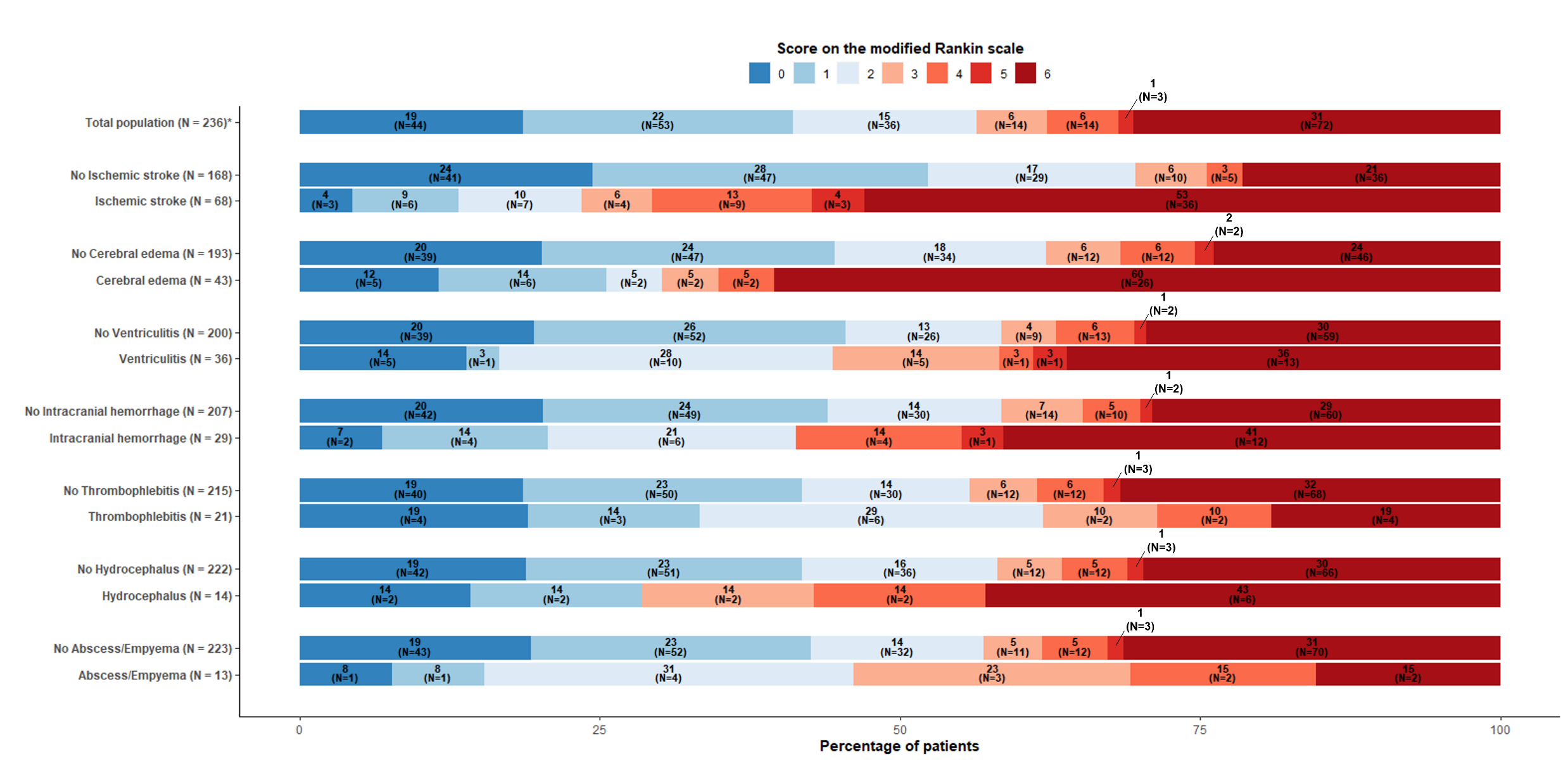
eFigure 4 – Distribution of modified Rankin scale according to intracranial complications among 236 patients *

**No cerebral venous thrombosis (N=215)**

**Cerebral venous thrombosis (N= 21)**

* modified Rankin score was missing for one patient

# eFigure 5 - Distribution of modified Rankin scale according to multivariate predictors of unfavorable neurological outcome on 236 patients.


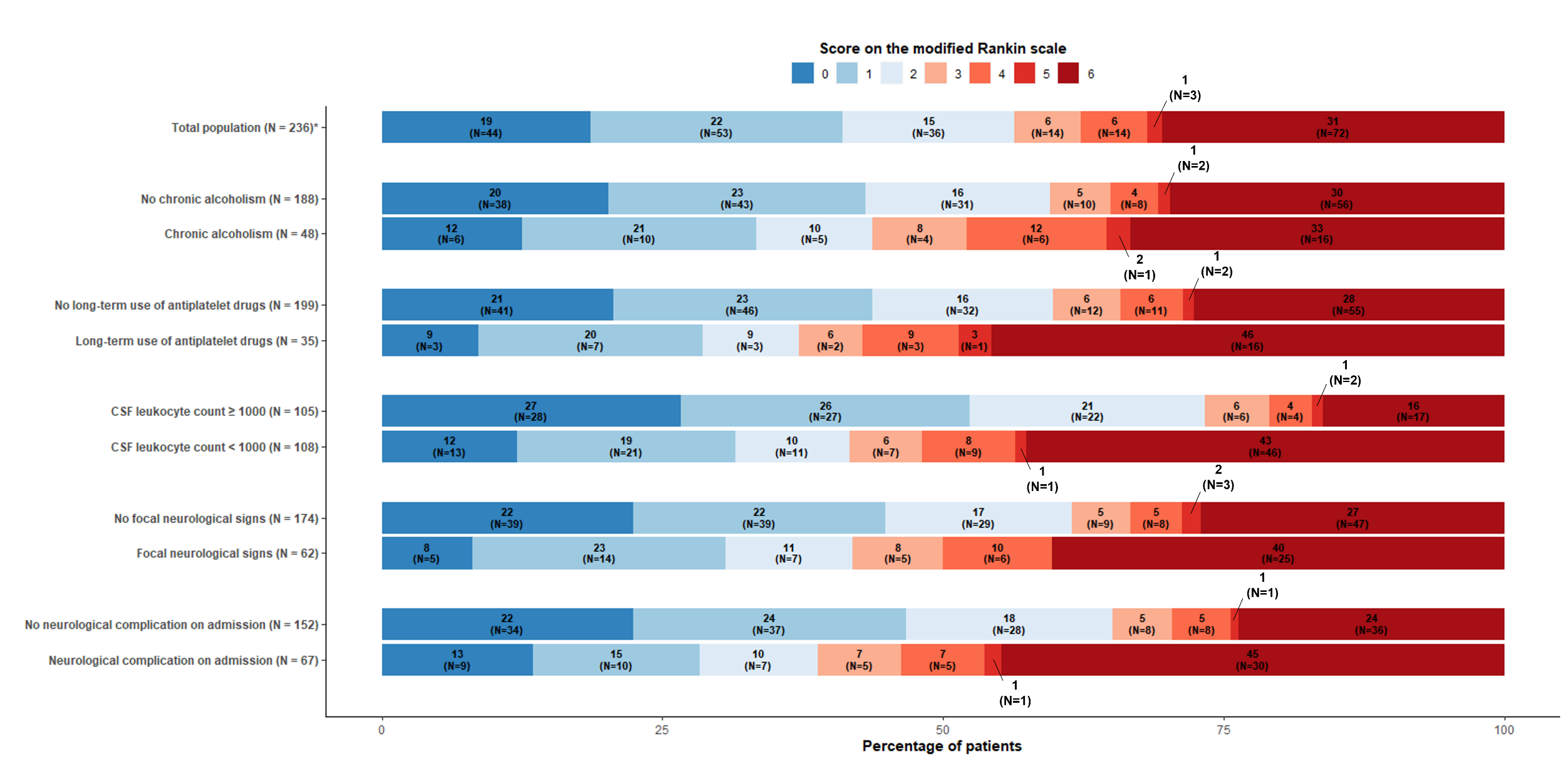


**Chronic vascular disease (N= 35)**

**No Chronic vascular disease (N= 199)**

***** modified Rankin score was missing for one patient
